# *“[Repeat] testing and counseling is one of the key [services] that the government should continue providing”*: participants’ perceptions on extended repeat HIV testing and enhanced counseling (ERHTEC) for primary HIV prevention in pregnant and lactating women in the PRIMAL study, Uganda

**DOI:** 10.1186/s12889-020-08738-x

**Published:** 2020-05-15

**Authors:** Femke Bannink Mbazzi, Zikulah Namukwaya, Alexander Amone, Francis Ojok, Juliane Etima, Josaphat Byamugisha, Elly Katabira, Mary Glenn Fowler, Jaco Homsy, Rachel King, the PRIMAL Study Team, Michael Adengo, Regina Adoo, Sandra Agondeze, Phiona Agwalo, Mirriam Ajok, Concy Akot, Chiara Akumu, Sharon Amama, Josephine Aparo, Pamela Atim, Joshua Buule, Lynae Darbes, Alice Elwana, Elizabeth Kakande, Elly Katabira, Ruth Kayondo, Grace Kezaabu, Beatrice Kibuuka, Max Kiwewa, Justine Lagol, Emily Likico Opu, Sarah Lunkuse, Joyce Matovu, Kenneth Mwambi, Josephine Nabukenya, Sarah Nakabuye, Noor Nakigozi, Gorrethy Nalubega, Esther Nambi, Halima Namukasa, Christine Namulwasike, Brenda Namusoke, Maria Nansasi, Irene Viola Nantongo, Vivian Ntono, Florence Ochan, Juliet Ogwang, Lawrence Ojom, Michael Okwera, David Oryema, Erick Otema, Betty Oweka, Joyce Rwechungura, Gertrude Sentongo, Jane Ssebaggala, Ruth Ssentongo, Anitah Wanyana

**Affiliations:** 1 Medical Research Council / Uganda Virus Research Institute & London School of Hygiene and Tropical Medicine Uganda Research Unit, P.O. Box 49, Entebbe, Uganda; 2 Faculty of Psychology and Educational Sciences, Ghent University, Ghent, Belgium; 3 Makerere University – Johns Hopkins University Research Collaboration, Kampala, Uganda; 4 AVSI Foundation, Kampala, Uganda; 5 Department of Obstetrics and Gynecology, Makerere University School of Medicine, Kampala, Uganda; 6 College of Health Sciences, School of Medicine, Makerere University, Kampala, Uganda; 7 Department of Medicine, Johns Hopkins University, Baltimore, MD USA; 8 Institute for Global Health Sciences, University of California, San Francisco, CA USA

**Keywords:** HIV, Prevention, Counselling, Repeat testing, Pregnancy, Postpartum, Acceptability, Feasibility, Uganda, Africa

## Abstract

**Background:**

The ‘Primary HIV Prevention among Pregnant and Lactating Ugandan Women’ (PRIMAL) randomized controlled trial aimed to assess an enhanced counseling strategy linked to extended postpartum repeat HIV testing and enhanced counseling among 820 HIV-negative pregnant and lactating women aged 18–49 years and 410 of their male partners to address the first pillar of the WHO Global Strategy for the Prevention of Mother-to-Child HIV transmission (PMTCT). This paper presents findings of qualitative studies aimed at evaluating participants’ and service providers’ perceptions on the acceptability and feasibility of the intervention and at understanding the effects of the intervention on risk reduction, couple communication, and emotional support from women’s partners.

**Methods:**

PRIMAL Study participants were enrolled from two antenatal care clinics and randomized 1:1 to an intervention or control arm. Both arms received repeat sexually transmitted infections (STI) and HIV testing at enrolment, labor and delivery, and at 3, 6, 12, 18 and 24 months postpartum. The intervention consisted of enhanced quarterly counseling on HIV risk reduction, couple communication, family planning and nutrition delivered by study counselors through up to 24 months post-partum. Control participants received repeat standard post-test counseling. Qualitative data were collected from intervention women participants, counsellors and midwives at baseline, midline and end of the study through 18 focus group discussions and 44 key informant interviews. Data analysis followed a thematic approach using framework analysis and a matrix-based system for organizing, reducing, and synthesizing data.

**Results:**

At baseline, FGD participants mentioned multiple sexual partners and lack of condom use as the main risks for pregnant and lactating women to acquire HIV. The main reasons for having multiple sexual partners were 1) the cultural practice not to have sex in the late pre-natal and early post-natal period; 2) increased sexual desire during pregnancy; 3) alcohol abuse; 4) poverty; and 5) conflict in couples. Consistent condom use at baseline was limited due to lack of knowledge and low acceptance of condom use in couples. The majority of intervention participants enrolled as couples felt enhanced counselling improved understanding, faithfulness, mutual support and appreciation within their couple. Another benefit mentioned by participants was improvement of couple communication and negotiation, as well as daily decision-making around sexual needs, family planning and condom use. Participants stressed the importance of providing counselling services to all couples.

**Conclusion:**

This study shows that enhanced individual and couple counselling linked to extended repeat HIV and STI testing and focusing on HIV prevention, couple communication, family planning and nutrition is a feasible and acceptable intervention that could enhance risk reduction programs among pregnant and lactating women.

**Trial registration:**

ClinicalTrials.gov registration number NCT01882998, date of registration 21st June 2013.

## Background

Mother-to-child transmission (MTCT) of HIV in sub-Saharan Africa is the leading cause of pediatric HIV infections [1]. Identification and treatment of HIV-infected mothers and preventing the vertical transmission of HIV has significantly reduced MTCT since the inception of prevention of MTCT (PMTCT) efforts in 1999 [2, 3]. The majority of pregnant women who test for HIV through national PMTCT programs are HIV-uninfected. Keeping these women uninfected throughout pregnancy and lactation is the first pillar of the Global World Health Organization (WHO) PMTCT strategy [4] and a key component to eliminate MTCT [5]. However, few national PMTCT programs have made the primary prevention of HIV acquisition a priority.

Studies in East and Southern Africa have shown that couple HIV testing and counseling (CHTC) increases uptake of HIV prevention and care services and identification of HIV sero-discordant couples [6–8]. In Uganda, HIV testing is routine in antenatal care (ANC) facilities in the country with 96% of women tested at health facilities in 2017 [9]. Partner involvement continues to be challenging, especially in the capital city Kampala with 12% coverage, whilst uptake of HIV testing by partners in the northern region where Kitgum district is located is much higher with 70% [9]. HIV Counselling and Testing (HCT) services are widely available in the country, and standard counselling is offered to individual women and women with partners in all ANC clinics following national guidelines [10]. For all HIV-positive women and partners, ART treatment and counselling on positive living is equally available at most health facilities and various outreach programs have been established [9, 11]. Pregnant women who test HIV-negative at first ANC visit may be retested at the time of labor and delivery if assessed to have been at risk. To our knowledge however, no specific program or counseling and retesting guide was in place at the time of the PRIMAL study to keep HIV-negative pregnant women negative throughout pregnancy and lactation. Existing maternal and child programs during antenatal and postnatal care for HIV-negative women were mainly focused on infant feeding, immunization and family planning [12].

The ‘Primary HIV Prevention among Pregnant and Lactating Ugandan Women’ (PRIMAL) randomized controlled trial (RCT) assessed the effect of the enhanced counseling component effects of an extended repeat HIV testing and enhanced counseling (ERHTEC) intervention (described in Methods below) on 410 HIV-negative pregnant and lactating women and 205 of their male partners compared to an equal number of controls on risk reduction behavior and the risk of sexually transmitted infections (STI) and HIV acquisition by these mothers during late pregnancy and throughout the breastfeeding period [9]. In each arm of the trial, half of the participants were enrolled individually and the other half were enrolled with their partner (as couples) based on their own spontaneous presentation at the ANC clinic. The research hypotheses were that; 1) ERHTEC during late pregnancy and throughout breastfeeding can increase and sustain risk reduction behaviors and prevent incident STI and HIV infections among HIV-uninfected pregnant women, and 2) that couple HIV testing and counseling (HTC) can further enhance this effect through improved couple communication and emotional and economic support from male partners.

The quantitative results of the RCT showed that condom use increased during follow-up but not significantly differently between the intervention and control arms of the trial, and that the incidence of HIV and STIs (syphilis, gonorrhea, chlamydia and trichomoniasis) among this cohort remained very low during follow-up in both the intervention and control groups (also not significantly differently) in contrast to comparable population-based Ugandan cohorts [9].

In addition to the published quantitative results of the RCT on the frequency of condom use and the incidence of HIV and STIs over follow-up [9], we conducted and report here the findings of qualitative components of the PRIMAL Study that were conceived as part of the initial study protocol. This paper thus describes PRIMAL participants’ experiences with and acceptance of the intervention and the perceived effects of the intervention on risk reduction, couple communication, and emotional support from partners.

## Methods

### Study population

#### Parent study

The PRIMAL study and its quantitative outcomes have been described in detail elsewhere [13]. Briefly, it enrolled 820 HIV-negative pregnant women aged 18–49 years with intention to breastfeed and 410 of their male partners from the antenatal care (ANC) clinics of Mulago National Referral Hospital in Uganda’s capital city of Kampala, and of St Joseph’s Hospital in the small rural town of Kitgum, Northern Uganda, 430 kms north of Kampala.

Women enrolled individually and enrolled couples were randomly assigned 1:1 to the ERHTEC intervention or a control group who received standard ante- and post-natal counseling linked to repeat testing. Both groups were followed up to 24 months postpartum or 6 weeks after complete cessation of breastfeeding, whichever occurred first.

#### Qualitative study

All participants of this qualitative study were part of the intervention arm and were purposefully selected in order to understand the experiences of men and women pertaining to these different categories. Purposive selection for KIIs and FGDs was conducted to ensure different categories of participants were represented, including pregnant women, breastfeeding women, women who had stopped breastfeeding, women who had a second pregnancy during the study, and male partners. Homogeneity of FGD participants was ensured by checking socio-demographic data prior to selection. At baseline FGD participants were recruited during routine clinic visits. At mid-term and end-of-study participants were requested to participate in FGDs and KIIs during a study clinic visit or by phone contact. Health workers including study counsellors, nurses/midwives and laboratory assistants were also interviewed through separate FGDs and KIIs as they all interacted with study participants.

### Intervention

Both intervention and control participants received repeat STI and HIV testing at enrolment, around labor and delivery, and at 3, 6, 12, 18 and 24 months postpartum. Women and couples assigned to the control arm of the study received standard post-test counseling as per current national guidelines delivered by available antenatal clinic staff only at the time of post-test HV counseling. Individual women and couples enrolled in the intervention arm received enhanced HIV prevention counseling every 3 months throughout follow-up delivered by certified study counselors trained in the ERHTEC intervention.

Enhanced HIV prevention counseling was based on a standardized guide developed for the study. The guide was developed following a workshop organized to train PRIMAL counselors on the intervention in July 2012 and updated thereafter as per these counselors’ experiences and recommendations. Study investigators selected themes relevant to the main research questions of the study based on findings from baseline formative research [14], including primary HIV and STI prevention; family planning; infant and young child feeding and nutrition (IYCF); counseling, communication and negotiation skills, and behavior change. The three-day counseling workshop was facilitated by experts in these areas. The goal of the training was to orient the PRIMAL study coordinators (2), research assistants (4), and study counselors (6) on the ERHTEC intervention and equip them with the knowledge and skills required to deliver ERHTEC. The topics and issues discussed in this training were used to inform the content and structure of the enhanced counselling guide including cue cards, summarizing key steps and messages inherent to each step of the EHRTEC intervention. As well, IEC materials were developed to guide the main topics of the counseling session.

**Fig. 1:**
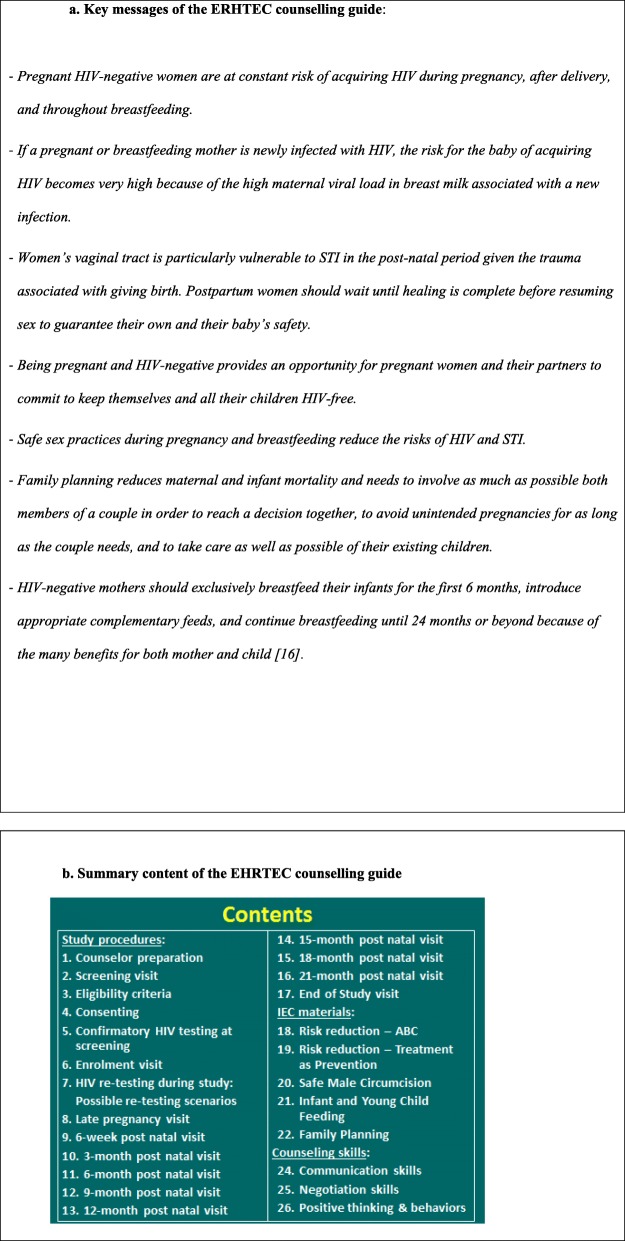
**a** Key messages of the ERHTEC Counselling Guide. **b** Summary content of the EHRTEC Counselling Guide

The counseling guide addressed the specific contexts and risks of incident infection in pregnant and lactating women. In addition to integrating the above guidance aimed at all pregnant and breastfeeding women according to national guidelines [15, 16], the guide clarified the concepts of acute and incident infection, explained the “window” period of HIV testing, addressed risk behaviors and sources of potential exposure to HIV between HIV tests, specific vulnerabilities and risks associated with different stages of pregnancy, delivery and post-partum periods including cultural practices and beliefs associated with sexual activity during pregnancy and breastfeeding, and resumption of sexual activity after delivery. It also discussed sero-discordance, its implications and all available ways to minimize transmission risks as well as disclosure, communication, sexual behavior, prophylaxis and care issues relevant to sero-concordant and sero-discordant couples. Finally, it included a section specific to couples in accordance with the guidelines currently in use in Uganda for couple HTC [17, 18], including for couples becoming newly sero-discordant. Study counsellors were trained on using marriage counselling techniques when addressing communication and negotiation challenges in couples, and cognitive behavioral therapy methods when challenging negative thinking and participant behavior. Figure 1a and b display the key messages and content of the counselling guide.

Case discussions and technical supervision were provided to the six study counsellors on a monthly basis by five senior counselling and psychotherapy supervisors with extensive work experience with individual clients and couples in Uganda. The supervisors were based in Kampala and travelled to the Kitgum site for supervision visits on rotational basis. During these visits, senior counselling supervisors provided mentoring through sit-in sessions throughout the study.

### Data collection and analysis

In January 2013, 6 focus group discussions (FGDs) were held to collect baseline information from the following discrete groups of participants: 8 pregnant and lactating women in Kampala, 9 in Kitgum; 7 male partners enrolled in the study in Kampala, 10 in Kitgum; 8 study health workers in Kampala and 9 in Kitgum. In April 2014, 6 mid-term FGDs were held with the following discrete groups of intervention arm women participants and study health workers: 8 women in Kampala, 9 in Kitgum; 9 enrolled male partners enrolled in the study in Kampala, 10 in Kitgum; 9 health workers in Kampala and 9 in Kitgum. At the end of the study, between July and September 2015, 44 key informant interviews (KIIs) and 6 FGDs were held with participant women enrolled in the intervention arm to evaluate the intervention. The 6 end-of-study FGDs were held with: 8 women enrolled individually in Kampala, 6 in Kitgum; 8 women enrolled in a couple in Kampala, 9 in Kitgum; 11 enrolled male partners of these women in Kampala and 10 in Kitgum. End of study KIIs were held with 4 discordant couples (3 in Kampala, 1 in Kitgum), 10 women who had stopped breastfeeding by the end of their follow-up (5 in Kampala, 5 in Kitgum), 9 women who were still breastfeeding by the end of their follow-up (5 in Kampala, 4 in Kitgum), 10 enrolled male partners of these women (5 in Kampala, 5 in Kitgum), 10 women with recurrent pregnancies by the end of their follow-up (5 in Kampala, in Kitgum), and one seroconverted woman enrolled individually (in Kitgum).

Investigators participated in drafting the KII and FGD guides, which consisted of questions around HIV, primary prevention, testing and counselling, and more specifically on the ERHTEC intervention at endline. FGDs were moderated by two female Ugandan Counselling Supervisors, trained and experienced in qualitative data collection in HIV studies in the two regions. Both are Ugandan nationals fluent in either Acholi, the language spoken in Kitgum district, or Luganda, the language spoken in Kampala city. KII interviewers were trained, overseen and continuously mentored by investigators. FGD moderators were aided by a note taker. Quality checks were conducted by the investigators but none of the investigators or research team implementing the intervention participated in FGDs or KIIs in order to prevent biased responses. On average, KIIs lasted 45 min while FGDs lasted 80 min. All KIIs and FGDs were audio recorded, transcribed verbatim, and translated by the research team. Participants had all consented to these procedures and received information about this study and the researchers at the time of enrolment.

Analysis of KII and FGD data was carried out using Nvivo10 (QSR International, Melbourne, Australia). Data was reviewed following a thematic approach using framework analysis, and a matrix-based system for organizing, reducing, and synthesizing data [19]. A codebook was developed by two investigators and imported into NVivo 10. The thematically organized data were then reviewed and synthesized into meaningful themes and quotes were selected to highlight, explain or describe relevant themes. Data saturation was discussed by the analysis team and informed the number of FGDs and KIIs conducted.

## Results

Table 1 summarizes FGD and KII participants’ characteristics. Age range was 18 to 44 years. The majority of Kitgum participants were peasant farmers, while in Kampala most were self-employed. Themes identified at baseline focused on risk reduction factors. Midline and endline themes focused on the acceptability and impact of the intervention, as described below.

**Table 1:** Characteristics of participants of focus groups discussions and key informant interviews

Time	Focus group discussions	Key Informant Interviews
Baseline January 2013	6 FGDs (3 in Kampala, 3 in Kitgum) with: - 17 pregnant and lactating women - 18 male partners of pregnant and lactating women - 17 health workers	None
Mid-study April 2014	6 FGDs (3 in Kampala, 3 in Kitgum) with: Kampala: - 4 individually enrolled women - 4 women enrolled in couple - 9 men enrolled in couple - 9 health workers Kitgum - 5 individually enrolled women - 4 women enrolled in couple - 10 men enrolled in couple - 9 health workers	None
End of study July–September 2015	6 FGDs (3 in Kampala, 3 in Kitgum) with: Kampala 8 individually enrolled women 8 women enrolled in couple 11 men enrolled in couple Kitgum 6 individually enrolled women 9 women enrolled in couple 10 men enrolled in couple	44 KIIs with: Kampala 3 women in discordant couples 5 women who stopped breastfeeding 5 women who were still breastfeeding 5 men enrolled in couple 5 women with recurring pregnancies Kitgum 1 woman in discordant couple 5 women who stopped breastfeeding 4 women who were still breastfeeding 5 men enrolled in couple 5 women with recurring pregnancies 1 woman who seroconverted

### Baseline

**Fig. 2:**
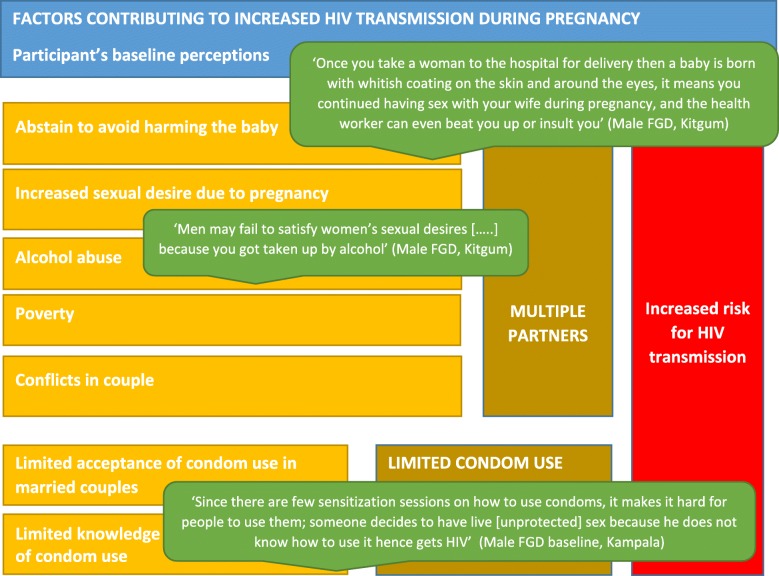
Conceptual Framework at baseline: Factors contributing to increased HIV transmission during pregnancy

Only FGDs were conducted at baseline. Women, men and health worker participants explored themes around pregnancy and HIV transmission, sexual behavior and risk of HIV transmission, and the role of men in HIV prevention. They identified factors, displayed in Fig. 2, which increase the number of sexual partners and limited use of condoms, and result into increased risk of HIV transmission in pregnancy.

#### Factors related to multiple partners

In both study sites, FGD participants were unaware of the increased biological vulnerability of pregnant and lactating women and their children to HIV transmission. FGD results showed that culturally, pregnant women were expected to sleep in separate residences from their husband in late pregnancy and early postnatal period. This practice is related to the view that the baby will be born ‘dirty’ and might be more vulnerable to illness or death if couples have sexual intercourse during this period. This forced abstinence was however mentioned as an increased risk for men to seek outside partners. In turn, the increased sexual desire of women during pregnancy was mentioned by the male focus groups as a risk for women to look for other partners if her husband abstains for the reasons mentioned above. All participants highlighted factors indirectly affecting HIV risk including poverty, conflict between couples, and alcohol use. Alcohol use was said to increase the risk of HIV transmission through having unprotected sex with multiple partners. However in the male FGD, men mentioned that when drinking alcohol the risk would reduce as the men would not be able to ‘perform’ and hence no sexual intercourse would take place. This in turn could leave the women sexually unsatisfied and lead to women looking for other sexual partners.

#### Factors related to condom use

FGD participants had knowledge of HIV prevention methods including testing, abstinence, male circumcision, being faithful and condom use. Participants admitted not to be fully conversant with condom use as how to use them is not always taught in sensitization sessions. Pregnant women and their partners mentioned that using condoms consistently was particularly difficult if both tested HIV-negative; condoms were perceived as something only discordant couples would use. Most men stated they had never used condoms while women stated that they did not use condoms while pregnant as they perceive condoms as a family planning method. Male partners emphasized how they should be respectful of their wives, show love and satisfy their wives’ sexual, emotional and material needs during pregnancy and lactation. Male participants also mentioned they would be willing to test themselves and other partners for HIV, and be treated any STIs.

### Midline

Midline FGD themes centered on the experiences of intervention participants with the PRIMAL study and EHRTEC intervention, acceptance and use of condoms, participants’ perceptions about their own risk for infection, the role of the male partner in prevention, and family planning awareness and use. Participants felt the EHRTEC intervention was helpful in improving trust and communication in couples, increasing knowledge on condom use, family planning, nutrition, and easing access to services in the hospital. Participants enrolled as couples appreciated couple counselling.

*“When you come with your husband, the counselor can involve both of you. In this situation, both of you are free to talk about issues that you are unable to talk about at home. At home, you may tell him about what he is doing wrong or he may try to talk to you about something which annoys him but in the presence of the counselor, you are both able to talk about that and at the end of the day, you go back home happy.”*

*Female participant, pregnant, FGD, Kampala*

Despite counselling on using condoms for risk reduction, both male and female participants were still apprehensive about its use in marriage. Condom use was generally limited to a few months after delivery for family planning purposes. Condoms were favored over other modern family planning methods as many said to have experienced side effects of oral contraception, injection and implant methods.

*“We had to use condoms because my wife had very bad side effects of other family planning.”*

*Male participant, FGD, Kitgum*

Female participants and health workers also felt EHRTEC increased male involvement in antenatal and postnatal care. Male participants mentioned the intervention had made them more faithful to their wives.

*“PRIMAL has brought a lot of peace to my house [...] through counselling, we are a lot happier and trust each other [...] I have now resolved to remain faithful to my wife”*

*Male participant, FGD, Kitgum*

### End-of-study

#### Acceptability and participant’s perceptions of EHRTEC intervention

Overall intervention participants expressed their satisfaction with the ERHTEC services. Participants gave positive feedback about the repeat testing and enhanced counselling intervention, and felt supported by the EHRTEC counselors in numerous aspects of their lives.

*“I have been able to open up my heart to the person counseling me, I feel like my heart has been relieved of a burden”*

*Female participant, enrolled individually, postpartum, KII, Kampala*

*“In PRIMAL you would see a counselor you could discuss anything with them because we were free with them, anything and I mean anything”*

*Female participant, enrolled individually, postpartum, KII, Kitgum*

Participants also appreciated testing and treatment for STI in addition to HIV, something which is not done in general antenatal care unless requested and paid for by the patient.

*“Whenever we visit, you test us for HIV or any other STDs. This has a great benefit because not every mother thinks of testing after every 3 months or after one month [ … .] It is very different from the other ordinary antenatal services”*

*Female participant, enrolled in couple, pregnant, KII, Kitgum*

The frequent telephone reminders to come for follow-up appointments and home visits in case of missed appointments were regarded as a positive part of the intervention.

*“The reminders were useful because they called me and that helped to prepare and they also made me think about testing and knowing my status.”*

*Female participant, enrolled individually, postpartum, KII, Kitgum*

Some study couples in Kitgum mentioned participation in the study had increased their status as a couple in the village, and were pleased about this.

*“We are being consulted by people in our community; 2-3 people contact us each time for advice and we talk to them because they now admire our relationship, they see us going to hospital together and even both of us take responsibility in looking after our baby if my wife is busy I help her out.”*

*Male participant, FGD, Kitgum*

#### Benefits of ERHTEC

**Fig. 3:**
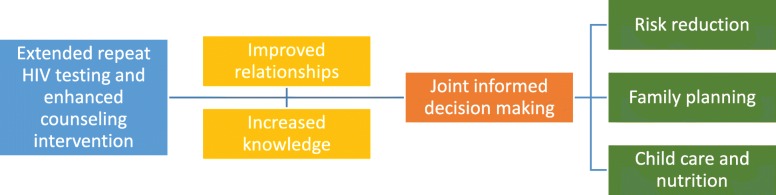
EHRTEC intervention benefits described by participants at end of study

The benefits mentioned in the interview and FGD end-of-study data are in line with the benefits mentioned at midterm. The majority of participants felt their relationship with their partner improved due to the ERHTEC couple counselling. EHRTEC counselling was reported to increase understanding among couples, improve communication, and result in more united decision-making. Participants reported that they acquired knowledge about HIV prevention, family planning and child spacing, taking care of a newborn, and infant feeding, which helped them make more informed decisions. Figure 3 describes the benefits participants described. Firstly participants felt the intervention had improved their couple and family relationships, and secondly they felt it increased their knowledge. This together improved decision making about risk reduction, family planning, and child care and nutrition.

##### Benefit ‘improved relationships’

EHRTEC counselling increased trust and faithfulness in couples’ relationships. Women and men enrolled as couples felt their partner was more supportive due to the counselling, and their relationships improved. Participants felt the counselling helped them solve problems together:

*“I have benefited through counseling. Usually if we have problems, we come here and get counseled then get back together and start afresh”.*

*Female participant, enrolled individually, postpartum, KII, Kampala*

*“We were counseled together and we were able to do everything together. We could discuss and we stopped fighting like we used to”*

*Female participant, enrolled in couple, pregnant, KII, Kitgum*

The counselling facilitated better understanding of the partner’s behavior too. Various couples described how they had less misunderstandings and arguments which reduced due to couple counselling and better communication between partners. One of the male participants gave an example of how his wife understood his love for dancing and is not out to see other women:

*“I used to like to go out dancing and this brought a lot of misunderstandings between me and my wife because she thought I had another woman [ … ] When the PRIMAL study started and we started counseling and testing together we started discussing issues and I reduced on the dancing. I sometimes go with her so that she knows it’s not women but I just love to dance.”*

*Male participant, FGD, Kitgum*

A large number of participants said the testing and counselling increased the love between them as they would come to the clinic together and discuss issues together during the counselling sessions and afterwards at home. One of the male participants’ in Kitgum district described it as follows:

*“PRIMAL has helped me and my wife to love each other even more because we come and test together and we are able to discuss issues together”*

*Male participant, FGD, Kitgum*

Male involvement in antenatal care improved in a number of couples too. Men became more involved in antenatal appointments, and would also come in for follow up sessions after delivery. One of the participants describes how his change in prioritization could lead to a quarrel with his employer, indicating how his family oriented behavior is not yet widely socially accepted:

*“Before, as an individual, I came at my convenience, but now, ever since I joined PRIMAL, whether or not it is convenient, I have to make sure I escort my wife when she pregnant. I remember a time I told my boss I have to give my family first priority even if he quarreled”.*

*Male participant, FGD, Kampala*

##### Benefit ‘increased knowledge’

Participants explained they had increased knowledge about HIV prevention, specifically HIV testing, faithfulness and condom use. They felt the counselling made them more thoughtful about having sexual affairs, and see the benefit of HIV testing. Two male participants described this as follows:

*“The counseling helps us not get HIV because when you go back home you recall what you were taught, you fear to go out and have other sexual affairs”.*

*Male participant, FGD, Kitgum*

*“When I joined the study, I realized that it was important to get tested”*

*Male participant, FGD, Kampala*

Participants described they learned how to use condoms, and discuss condom use with their partners during the counselling sessions. During the sessions the counsellors noted that whilst many people had heard about and seen condoms, many did not know how to practically and correctly use one. By discussing how to ensure availability, where to store condoms safely, demonstrating correct use, and discuss disposal, participants felt more knowledgeable.

*“They (study counsellors) taught us how to use condoms”*

*Female participant, KII, Kampala*

In addition participants described they increased their knowledge of family planning, child care and nutrition through participation in the EHRTEC intervention arm. Participants mentioned the financial and health benefits of family planning, and the importance of good infant feeding practices.

*“Couple counseling helped me to know our status [ … ], to know how I take care of my babies and how I space them [children], to know the number I am going to give birth to”*

*Female participant couple, KII, Kampala*

*“They taught us how to feed our baby, when to introduce porridge, then when to introduce other foods. They also taught the mother how to feed themselves so that they would have enough breast milk.”*

*Female participant, KII, Kitgum*

##### Benefit ‘Joint informed decision-making’ – risk reduction

The ERHTEC couple counselling helped participants to discuss and plan more together. This applied to domestic decisions, as well as understanding each others’ sexual needs and desires.

*“We respect each other even in sexual matters, I don’t have to have sex all the time”.*

*Female participant, seroconverted, KII, Kitgum*

*“When I feel I should have sex, I inform her. When she feels she should have sex, she informs me. Earlier it was my decision”*

*Male participant, KII, Kitgum*

The joined informed decision-making was noted in choices for HIV prevention, decisions around family planning, and child care and nutrition.

Participants appreciated the regular counselling as an HIV prevention method. They mentioned that counselling helped them understand and appreciate the benefits of HIV testing, risk reduction, and how to practice this at home.

*“I went home and he asked me what they had found. I said that I was [HIV-]negative but I will not have unprotected sex with him because I was not sure of his status. He also eventually went and tested and brought his test results [home].”*

*Individual woman, FGD, Kitgum*

Participants expressed there was a reduction in extramarital affairs in the couple due to counselling which helped them to prevent HIV transmission.

*“I have learnt to be faithful to my partner”*

*Female participant, enrolled individually postpartum, KII, Kampala*

*“My husband has changed he doesn’t have extra marital affairs anymore.”*

*Female participant, enrolled in couple, post partum, FGD, Kitgum*

None of the participants interviewed through FGDs or KIIs reported consistent condom use. Condoms were used for ‘short’ periods, e.g. only during pregnancy or when they wanted to prevent pregnancy. Participants placed more emphasis on how the intervention had changed their relationships and communication about sexual and reproductive health, and increased faithfulness.

*“This counseling has helped us to have safer sex, we can choose to use condoms or abstain”*

*Male participant, KII, Kitgum*

*“We trust each other so we use the condoms just for family planning but not for HIV prevention”*

*Female participant, enrolled in couple, postpartum, FGD, Kitgum*

##### Benefit ‘Joint informed decision-making’ – family planning

The counselling sessions provided information on family planning, and facilitated discussions between members of the couple on its use. Individual women mentioned the challenge of discussing and making male partners accept the use of family planning methods.

*“It all depends on the kind of understanding you have in the home. Because you can tell the man that you are not yet ready to have another child but he would not listen. People who are able to space children are able to discuss as a couple and the man is willing to listen to what the woman has to say and the woman is also willing to hear what the man has to say”.*

*Female participant, enrolled individually, postpartum, KII, Kitgum.*

##### Benefit ‘Joint informed decision-making’ – child care and nutrition

Participants reported they received information on child care and nutrition. They felt this helped them to stay healthy and ensure their infant would develop well.

*“The information I got from PRIMAL helped me to eat the right food because of the education I got and also to feed my baby well and my baby is doing very well.”*

*Female participant, enrolled in couple, postpartum, FGD, Kitgum*

### Future recommendations regarding the ERHTEC intervention

Participants were asked whether they preferred couple or individual counseling and the majority in both sites had a preference for couple counselling. Those enrolled as individual women expressed the importance of having their partners involved, however they had not been able to enroll as couples mostly due to logistic challenges, (e.g. the husband was working far from his wife or having a job from which it was difficult to get permission to leave).

*“When you join as an individual you are learning alone, the information and even counseling are received by the woman alone and sometimes when you go back home to tell the man, he doesn’t want to listen; if he goes out and has an affair he will bring you the infection as well”.*

*Female participant, postpartum, KII, Kitgum*

In Kampala, participants recommended the combination of individual and couple counselling sessions:

*“They are both ok because as an individual, they will ask you only your personal concerns and then the doctor advises you on how you can manage, and when you talk as a couple, it creates a bridge”.*

*Male participant, KII, Kampala*

Participants recommended that the EHRTEC intervention be availed to all couples attending antenatal care, both in urban and rural settings.

*“The PRIMAL team should continue with this program because it is making our relationships better. They should continue with us and even have the program all over Uganda so that everyone benefits.”*

*Male participant, FGD, Kitgum*

*“Routine [repeat] testing and counseling is one of the key issues that the government should continue providing”*

*Male participant, KII, Kampala*

In Kitgum, participants requested for expansion of the services to lower level health facilities.

*“Extend the services because in the villages there are other people who cannot access such hospitals”.*

*Female participant, stopped breastfeeding, KII, Kitgum*

## Discussion

The EHRTEC intervention offered a comprehensive HIV risk reduction model that incorporated cultural, individual and dyadic dimensions of HIV risk and prevention specific to pregnant and lactating women. This qualitative study showed that the ERHTEC intervention is an acceptable and helpful intervention during and post pregnancy. Participants perceived that the intervention had positive effects on risk reduction, couple communication, and increased emotional support from partners.

PRIMAL study intervention arm participants felt the study helped them to improve their relationships, as well as increase knowledge about HIV prevention, family planning, and child care and nutrition. The counselling increased understanding among couples, improved communication, and resulted in more joined decision-making including negotiation about sex and condom use, family planning and child care and nutrition. Although no significant differences were found in the quantitative study in either condom use increase or in HIV and STI incidence over follow-up, the low annual HIV incidence rate of 0.278 per 100 women years of follow-up that characterized the entire study cohort of men and women over an average of 21 months of follow-up is in line with the risk reduction effect participants perceived through the EHRTEC intervention [13].

Couple counseling has its origins in marriage counselling in which couples were prepared for marriage individually and as a couple by family members, religious leaders, and counsellors [20]. The aim is to facilitate the development of a healthy relationship between partners with good couple communication and support [21]. In Uganda, couple HIV counseling and testing (CHCT) has been part of the National HIV Guidelines since 2005 [17, 18]. While these guidelines include a section on basic counselling skills, and address couple relationship, HIV testing, disclosure, and positive living, there is no emphasis on issues specific to pregnancy and the post-natal period, or on strengthening relationships between partners, improving communication, family planning, and nutrition. We suggest that HIV counselling continues to discuss risk reduction and positive living, and also focuses on couple communication to support joint decision-making for risk reduction in the particular contexts of pregnancy and breastfeeding.

Participants in our study mentioned that counseling helped them improve their faithfulness to their partner. They described this in context of having a better relationship, being able to trust each other more, and being able to communicate better. Faithfulness is promoted as the second pillar of the HIV Abstinence, Be faithful, use a Condom (ABC) strategy since the 1990s [22]. The ABC approach assumes that knowledge influences attitudes and results in behavior change accordingly [23], which we know is rarely the case [24]. Life skill interventions such as the Stepping Stones have tried to integrate HIV prevention into a broader discussion of reproductive health [25]. The EHRTEC intervention discussed social and cultural expectations and challenges of enrolled couples in relation to HIV prevention. These addressed expectations around abstinence in the last months of pregnancy due to cultural beliefs about harming the unborn baby. This may have facilitated better couple relationships, and more faithful behaviors.

This qualitative study showed that participants did feel more knowledgeable and empowered to negotiate sex and condom use. While the PRIMAL study did not find a significant statistical effect of the ERHTEC intervention in increasing condom use or in reducing HIV or STI incidence among HIV-uninfected pregnant and lactating Ugandan women, condom use did increase significantly over follow-up in both arms and both groups (individually enrolled women or couples) of the study [13]. This is in line with earlier reports on a slow increase of condom use amongst married couples in Uganda [26–28]. Counselling has also been found to increase condom use in sero-discordant couples [29]. Williamson et al. (2006) described that while Ugandan men in Kampala expressed a dislike for condoms initially, men’s perceptions of condoms tended to improve when they used them more frequently and started to feel more comfortable and confident in using them [27]. Pleasing their partners by using condoms was also important to the men in Williamson’s study. Thus, taking into consideration other motivations is important in strengthening HIV prevention strategies in antenatal and postnatal care.

Pre-exposure prophylaxis (PrEP) was not part of Uganda’s HIV prevention recommendations at the time of this study. Today it is recommended for HIV-uninfected individuals at high risk of acquiring HIV but implementation is limited due to restricted access to PrEP drugs in public health facilities [11]. The use of enhanced testing and counseling in conjunction with extended repeat HIV testing could be a complementary way to reinforce prevention in prenatal and postnatal care. PrEP has proven a safe and effective HIV prevention method for discordant and HIV-negative couples both during pregnancy and lactation [30–32]. Further counselling on the risk of HIV transmission during pregnancy and the benefits of the various options for HIV prevention including PrEP will be key to assist a person to make an informed choice and use prevention methods correctly.

The recommendation of participants to provide couple counselling and testing at all ANC services, with an emphasis of couple counselling and testing, supports the national couple counselling guidelines and WHO strategy [4, 11, 17]. The literature on HIV sero-discordant couples has shown that couple counselling and testing is associated with positive outcomes including reduced HIV transmission and unprotected sex, and increased rates of status disclosure and levels of treatment adherence [33]. Rosenberg et al. (2016) has shown that couple HIV counseling and testing (HCT) is more protective than individual HCT for HIV acquisition [34]. Nannozi et al. (2017) reported that male involvement in antenatal care is a motivating factor for CHCT [35]. The men in our study appreciated their involvement in ANC. While ERHTEC counselling sessions focused on risk reduction, sufficient time was allocated to child bearing, upbringing, nutrition and family planning. In South Africa and Tanzania, Yende et al. (2017) and Jefferys et al. (2015) found that invitation letters with focused messages on fatherhood in primary health care were motivating and promoted male involvement in ANC [36, 37]. Invitation letters have also been tried in Uganda with limited results [38, 39]. Better results have been obtained through sensitization of men by peers, making ANC and labor and delivery and maternity clinics and wards more ‘men-friendly’, offering CHCT services as standard of care, prioritizing couples for services, and approaching men about the role they can play in antenatal and postnatal care and for primary HIV prevention compared to a sole focus on HIV testing and counselling [40–42]. When communication in a couple improves, men and women may be more satisfied with their relationship which could reduce the number of sexual partners, and promote safer sex negotiation.

As Nduna et al. (2015) have argued, the epidemic has changed and while initially successful, the ABC prevention strategy may not be sufficient anymore. We know this is definitely true for serodiscordant couples but we need to look into an enhanced new primary MTCT prevention model in the context of multipronged interventions for both HIV discordant and seronegative concordant couples [43]. From the EHRTEC experience, such model may be include further male involvement in ANC, and enhanced individual and couple counselling with a focus on improving couple communication to negotiate for safer sex and condoms, in addition to new available prevention methods such as PrEP for serodiscordant couples or couples not knowing each other’s HIV serostatus.

### Strengths and limitations

The main strength of this study is that the PRIMAL study was the first randomized controlled study looking at primary prevention of HIV acquisition for up to 24 months postpartum in lactating women in sub-Saharan Africa. Another strength of the study was the inclusion of participants from a remote rural post-conflict site in Northern Uganda: most studies focused on antenatal HIV testing and couple counselling in Uganda have focused on urban centers and districts in Central, East, South and Western Uganda [29, 35, 44–47].

The main limitation of this study is that we did not collect similar data from control participants during the mid-term and end-of-study FGDs and KIIs. The rationale for this is that controls were not exposed to the enhanced counseling intervention which was the focus of this study. However, this prevents us from knowing if there was any difference in acceptability and perceived quality of extended repeat testing and counseling services between ERHTEC and control participants.

This study did not assess the effect of mental health symptoms in participants on the intervention. While the study addressed the risk of alcohol abuse with the couples, and referred those with a significant abuse history, support services for those with addiction are very limited in the country. This is especially relevant in northern Uganda where alcohol abuse is common due to high levels of trauma due to 25 years of war displacement between 1984 and 2006 [48, 49], and a chronic lack of rehabilitation programs.

## Conclusion

This qualitative study shows that pregnant and lactating women who received the ERHTEC intervention perceived that the intervention was acceptable and contributed to risk reduction and improved support, communication and decision-making about sexual and reproductive health with their partners. Thus, repeat postpartum HIV and STI testing and enhanced individual and couple counselling focusing on HIV prevention, couple communication, family planning and nutrition intervention, could constitute an enhancing component of risk reduction programs.

## Data Availability

The datasets used and/or analyzed during the current study are available from the corresponding author on reasonable request.

## Abbreviations

ANC: Ante natal care
CHTC: Couple HIV testing and counseling
EMTCT: Elimination of mother to child transmission of HIV
ERHTEC: Extended Repeat HIV Testing and Enhanced counseling
FGD: Focus group discussion
HTC: HIV testing and counseling
HIV: Human immunodeficiency virus
IYCF: Infant and young child feeding
KII: Key informant interview
MTCT: Mother to child transmission of HIV
PMTCT: Prevention of mother to child transmission of HIV
PrEP: Pre-exposure prophylaxis (PrEP)
PRIMAL: Primary HIV Prevention in pregnant and lactating Ugandan women
RCT: Randomized control trial
STI: Sexually transmitted infection
WHO: World Health Organization
